# Association between sleep duration and osteoarthritis and their prevalence in Koreans: A cross-sectional study

**DOI:** 10.1371/journal.pone.0230481

**Published:** 2020-04-27

**Authors:** Yongkyu Cho, Boyoung Jung, Yoon Jae Lee, Me-riong Kim, Eun-Jung Kim, Won-Suk Sung, In-Hyuk Ha

**Affiliations:** 1 Jaseng Hospital of Korean Medicine, Gangnam-gu, Seoul, Republic of Korea; 2 Department of Health Administration, Hanyang Women’s University, Seongdong-gu, Seoul, Republic of Korea; 3 Jaseng Spine and Joint Research Institute, Jaseng Medical Foundation, Gangnam-gu, Seoul, Republic of Korea; 4 Department of Acupuncture & Moxibustion, Dongguk University Bundang Oriental Hospital, Seongnam-si, Republic of Korea; University of Tasmania, AUSTRALIA

## Abstract

**Objective:**

To determine the association of radiological and symptomatic osteoarthritis with sleep duration in a representative sample of the Korean population.

**Methods:**

Using data from the national cross-sectional fifth Korea National Health and Nutrition Examination Survey 2010–2012. Of the 16,528 participants in KNHANES-V, 8,918 were adults aged≥ 50 years who had completed the survey questions on sleep duration and osteoarthritis, and had diagnostic X-ray results. We evaluated the association between sleep duration as the primary predictor for osteoarthritis involving the hip, knee, and spinal joints. A complex sample logistic regression analysis was performed to adjust for the covariates.

**Results:**

Proportions of participants with total daily sleep duration of ≤6 hours, 7–8 hours, and ≥9 hours were 47.1%, 45.2, and 7.7%, respectively. The rate of osteoarthritis diagnoses in the ≤6 hours, 7–8 hours, and ≥9 hours of sleep duration groups was 24.1%, 17.6%, and 21.8%, respectively (p <0.0001). The odds ratios (OR) were significantly higher in the ≤6 hours of sleep group than in the 7–8 hours of sleep group (OR, 1.20; 95% confidence interval [CI], 1.03–1.39; p = 0.02), but no significant difference in the ≥9 hours of sleep group was found after adjusting the confounding variables. When we compared knee joint pain (Numeric Rating Scale 0 versus 1–10) in participants with grade 2–4 Kellgren-Lawrence (KL) classification after adjusting these same confounding variables, the ≤6 hours of sleep group (OR, 1.32; 95% CI, 1.10–1.58) and the ≥9 hours of sleep group (OR, 1.41; 95% CI, 1.03–1.95) showed significantly higher ORs.

**Conclusion:**

This study confirmed the significant association between sleep duration and osteoarthritis in adults aged ≥50 years. Participants’ positive for both radiological (KL grade ≥2) and symptomatic osteoarthritis showed a strong association between knee joint pain and not enough sleep duration.

## Introduction

Osteoarthritis (OA) is a chronic disease with major symptoms of joint pain, stiffness, edema, and reduced joint function [1]. The condition is characterized by cartilage degeneration, or functional impairment, in one or more joints [2]. It commonly occurs in the knees, hips, and joints of the hands and feet, and is more prevalent in middle and old age. The prevalence of OA in adults aged ≥50 years in Korea is estimated to be 14.3% [3]. Knee osteoarthritis (KOA), in particular, is a common degenerative arthritic disorder that affects approximately 10%–25% of individuals ≥50 years worldwide [4]. OA is recognized as an important health issue, as it has a high rate of disability, reduces the quality of life for patients and their families, and increases medical expenses by around $875.27 per person per year [5].

Several risk factors related to increased OA incidence have been identified, and include increased body mass index (BMI), history of knee trauma, Heberden’s nodes, female sex, old age, vigorous physical activity, and increased bone mineral density [6]. Cardiovascular disease is also associated with an increased prevalence of OA in both males and females [7]. Additionally, OA in the hands and knees tends to develop more readily in patients with diabetes than in those without [8], and mental health issues also influence the prevalence of OA [6]. Thus, OA has an association with metabolic disease and mental health factors.

Furthermore, several studies have demonstrated short sleep duration to be a risk factor for obesity, hypertension, glucose intolerance, and cardiovascular disease in the general population [9–11]. Increased sympathetic tone and activation of inflammatory pathways due to short sleep duration affect hypertension and cardiovascular disease [12, 13]. Wang, et al., revealed that older age, smoking, irregular meal patterns, limited physical exercise, poor mental health, and chronic diseases were positively associated with short sleep duration [14]. There have also been reports that sleep duration affects mental health [14, 15]. Thus, sleep duration and sleep disorders are associated with various metabolic diseases and mental health [16].

Based on the relationship between OA and sleep duration with metabolic disease and mental health, we hypothesized that there would be direct and indirect associations between OA and sleep duration. Moreover, individuals with knee or lower back pain are more likely to complain of sleep difficulties [17], which supports this hypothesis. Sleep onset and sleep maintenance are affected by knee pain [18], and other studies have reported that cognitive-behavioral therapy (CBT) focusing on sleep can significantly reduce pain in KOA patients [19]. Although there have been several attempts to examine the relationship between sleep duration and OA and the use of CBT in treatment methods, no large-scale study has investigated the relationship between sleep duration and OA in a representative sample of the Korean population.

Therefore, we studied the direct relationship between sleep duration and OA and evaluated the relationship between sleep duration and musculoskeletal pain. Additionally, we investigated the association between sleep duration and symptomatic OA (clinically significant pain and other symptoms) and radiological OA (diagnostically significant changes in X-ray images) using a complex sample design [20].

## Materials and methods

### Study population

**Fig 1:**
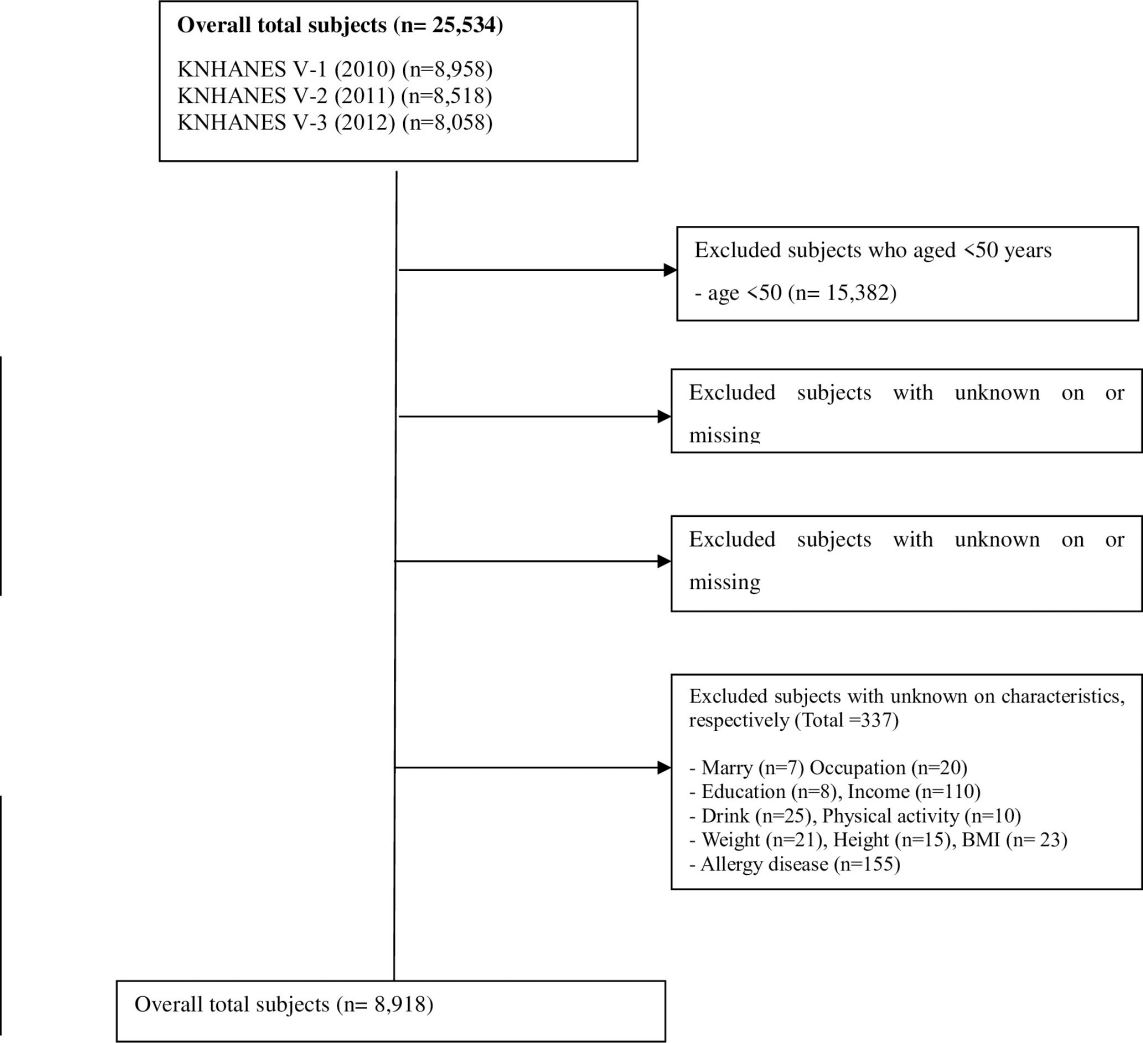
Subjects’ flow diagram.

Data were obtained from the fifth Korean National Health and Nutrition Examination Survey 2010–2012 (KNHANES-V), a nationwide survey conducted by the Korea Centers for Disease Control and Prevention. Using a stochastic sample of 23 households from 192 regions in Korea, around 10,000 household members aged ≥1 year were surveyed annually [21]. Participants were divided into life stages: children (1–11 years), adolescents (12–18 years), and adults (≥19 years), and the survey contents are adapted to each group. KNHANES consists of a household screening survey, health interview, health examination, and nutritional survey (https://knhanes.cdc.go.kr/knhanes/index.do). In this study, we used the 2010–2012 KNHANES-V data, which included OA radiography findings. Participant exclusion criteria included: (1) participants <50 years (n = 15,382); (2) missing sleep duration (n = 882); and (3) osteoarthritis data (n = 15); (4) other missing data (n = 337). Out of 25,534 participants in the KNHANES-V, 8,918 were adults aged ≥50 years who had completed the survey questions on sleep duration and OA and had diagnostic radiography results (Fig 1).

### Primary predictor: Evaluation of sleep duration

The sleep time used in this study was the average daily sleep time (time at which you go to bed and time at which you wake up) calculated in minutes. According to the National Sleep Foundation's sleep time duration criteria recommendations [22], we categorized sleep times as follows: short (≤6 hours of sleep per day), normal (7–8 hours of sleep per day), and long (≥9 hours of sleep per day). This standard was the same as reported in foreign precedent studies [23–25] and our previous studies [15, 26–28].

### Outcome and other variables

#### Evaluation of OA

OA was surveyed at three levels, based on questionnaire items, radiological diagnosis by X-ray, and joint pain. The questionnaire items included: lifetime OA (positive/negative), OA diagnosed by a doctor (yes/no), current OA (positive/negative), and currently receiving treatment for OA (yes/no). OA examination was performed by X-ray imaging of the hip, knee, and lumbar vertebral joints. **For the hip and knee joints,** a radiological diagnostic value was obtained using the Kellgren-Lawrence (KL) Grading Scale. The **hip joint** was graded as normal (0); suspected OA (1); mild OA (2); or moderate OA (3). **The knee joint** was graded as normal (0); suspected OA (1); mild OA (2); moderate OA (3); or severe OA (4). **The lumbar vertebral joints** were graded per the KL grades as normal (0); suspected OA (1); or OA (2). If participants had knee joint pain and knee joint KL grade ≥2, or hip joint pain and hip joint KL grade ≥2, then OA was considered significant [29].

#### Numeric rating scale (NRS)

Though pain is a multidimensional concept, the subjective intensity is probably the most frequently measured component in clinical practice. For joint pain, participants were asked to indicate whether they had experienced knee pain for at least 30 days in the last 3 months before the survey, as well as the pain intensity on a numeric rating scale from 0 to 10, where ((1–5) was mild; (6–7) was moderate; and (8) was severe).

#### Covariates

Participants’ demographic and socioeconomic characteristics included sex, age, education, household income, marital status, occupation, and area of residence. For their behavioral health characteristics, we included alcohol consumption, smoking status, physical activity, and BMI.

Among demographic and socioeconomic characteristics, education was categorized as: (1) elementary school graduation or below; (2) middle school graduation; (3) high school graduation; (4) college graduation or above. Household income was categorized according to household income quartiles: (1) low; (2) low-middle; (3) high-middle; (4) high. Marital status was categorized into: (1) unmarried; (2) married and cohabitating with spouse;, (3) bereaved/divorced/separated. Among behavioral health characteristics, smoking status was categorized as: (1) current smoker, for individuals who had smoked in their lifetime and were currently smoking; (2) past smoker, for individuals who had smoked at least 5 packs (100 cigarettes) in their lifetime but were not currently smoking; (3) nonsmokers, who have never smoked in their lifetime. Alcohol consumption was categorized as ‘drinker’ or ‘non-drinker’ based on whether or not the individual had consumed alcohol at least once per month during the last year. Occupation was categorized into ‘unemployed’ and ‘employed,’ while area of residence was categorized into two groups: ‘*dong*’ and ‘*eup/myeon*.*’* BMI (kg/m^2^) was categorized into three groups: Group 1 (0 < BMI <18.5); Group 2 (18.5< = BMI <25); Group 3 (BMI ≥25) [30] Physical activity was categorized as ‘yes’ or ‘no’ based on whether the individual had performed at least 30 minutes of moderate physical activity (enough to make them feel physically tired or slightly short of breath) at least once in the past week.

#### Statistical analysis

The KNHANES applies stratified cluster sampling and weighted values to a nationally representative sample, which is based on the reciprocal of the selection probabilities (*psu*, household), the inverse of response rate (household, subject), and a post-stratification factor, which provides age and sex-specific survey result estimates for the Korean population [21]. Therefore, we performed data analysis based on a complex sampling design with elements of stratification variables, clustering variables, and weights [31]. We calculated the mean, standard deviation, and standard error for the continuous variables, and the frequency and percentage (%) for the categorical variables. **Additionally, we performed a Rao-Scott chi-square test, or analysis of variance, to analyze the differences in participant characteristics relating to sleep duration and the presence or absence of OA.** Traditionally, Pearson's chi-squared test was mainly used to cross-analyze categorical data; however, this data analysis does not follow the distribution, as stratified cluster sampling requires the use of correlation between observations. In order to evaluate the association between sleep duration and OA, we performed multivariate logistic regression analysis under a complex survey design, which allowed us to adjust the complex sample design for covariates. We calculated the odds ratios (ORs) and 95% confidence intervals (CIs) by using the age and BMI covariates as continuous variables and all other covariates as categorical variables. The statistics package SAS V9.4 (SAS Institute Inc, Cary, NC, USA) was used for all data analyses, and two-tail P values <0.05 were considered statistically significant.

## Results

Participants reported long sleep duration (≥9 hours of sleep/day, 7.7%) the least when compared to the short (≤6 hours of sleep/day, 47.1%) or normal (7–8 hours of sleep/day, 45.2%) sleep duration. Females reported shorter sleep durations than males, and the participants in the short and long sleep duration groups were older than those in the normal sleep duration group. Fewer married participants were in the short sleep duration group than in the normal or long sleep duration groups, while bereaved/divorced/separated participants were more in the short sleep duration group. The long sleep duration group showed a higher percentage (19.7%) of current smokers, while the short sleep duration group showed a higher percentage of non-smokers. Furthermore, participants with an elementary school graduation education or lower, low household incomes, or living in rural areas (*eup* or *myeon* administrative districts). Participants with past and present experience of OA were surveyed using four items. The results showed that few of these participants experienced normal sleep duration and that they had larger number of short sleep duration compared to participants who had never experienced OA (Table 1).

**Table 1:** Characteristics of study participants.

Factors		Sleep duration						P-value
		Short (≤6 h/day)		Normal (7–8 h/day)		Long (≥9 /day)		
		N	%	N	%	N	%	
Total		4196	**47.1**	4037	**45.2**	685	**7.7**	
**Sex**								
	Male	1598	38.1	1895	46.9	324	47.3	<0.0001
	Female	2598	61.9	2142	53.1	361	52.7	
**Age (years)**	(Mean±SE)	63.0±0.2		60.9±0.2		64.3±0.5		
**Education**								
Elementary school graduation or below		2169	51.7	1693	41.9	415	60.6	<0.0001
Middle school graduation		669	15.9	756	18.7	112	16.4	
High school graduation		911	21.7	1053	26.1	120	17.5	
College graduation or above		447	10.7	535	13.3	38	5.6	
**Household income**								
	Low	1387	33.1	1133	28.1	296	43.2	<0.0001
	Low-middle	1081	25.8	1038	25.7	176	25.7	
	High-middle	843	20.1	867	21.5	136	19.9	
	High	885	21.1	999	24.8	77	11.2	
**Marital status**								
Unmarried		26	0.6	39	1.0	1	0.2	<0.0001
Married (cohabitating with spouse)		3119	74.3	3313	82.1	553	80.7	
Bereaved, divorced, separated		1051	25.1	685	17.0	131	19.1	
**Alcohol consumption**								
	Non-drinker	2510	59.8	2199	54.5	373	54.5	<0.0001
	Drinker	1686	40.2	1838	45.5	312	45.6	
**Smoking status**								
	Current smoker	568	13.5	694	17.2	135	19.7	<0.0001
	Past smoker	968	23.1	1066	26.4	181	26.4	
	Non-smoker	2660	63.4	2277	56.4	369	53.9	
**Occupation**								
	Unemployed	2165	51.6	1826	45.2	362	52.9	<0.0001
	Employed	2031	48.4	2211	54.8	323	47.2	
**Area of residence**								
	Dong	3131	74.6	2887	71.5	421	61.5	
	Eup/myeon	1065	25.4	1150	28.5	264	38.5	
**BMI (kg/m2)**								
	(Mean±SE)	24.1±0.1		24.0±0.1		23.6±0.2		
Underweight	(BMI<18.5)	109	2.6	108	2.7	35	5.1	0.01
Normal weight	(18.5≤BMI<25)	2598	61.9	2528	62.6	429	62.6	
Obese	(25≤BMI)	1489	35.5	1401	34.7	221	32.3	
**Physical activity**								
	No	3806	90.7	3674	91.0	635	92.7	0.29
	Yes	390	9.3	363	9.0	50	7.3	
**Lifetime OA**								
	No	2955	70.4	3149	78.0	503	73.4	<0.0001
	Yes	1241	29.6	888	22.0	182	26.6	
**OA diagnosed by a doctor**								
	No	3184	75.9	3325	82.4	536	78.3	<0.0001
	Yes	1012	**24.1**	**712**	**17.6**	**149**	**21.8**	
**Current OA**								
	No	3305	78.8	3438	85.2	552	80.6	<0.0001
	Yes	891	21.2	599	14.8	133	19.4	
**Currently receiving treatment for OA**								
	No	3695	88.1	3710	91.9	601	87.7	<0.0001
	Yes	501	11.9	327	8.1	84	12.3	

* Rao-Scott chi-square test was performed to determine the differences between groups.

Abbreviations: BMI, body mass index; OA, osteoarthritis; SE, Standard Error

Table 2 shows the relationship between sleep duration and KL grade by the type of joint. The lower the KL grade, the higher the percentage of participants with normal sleep duration. However, the higher the KL grade, the higher the percentage of participants sleeping ≤6 hours of sleep/day or ≥9 hours of sleep/day, with more participants sleeping for ≥9 hours of sleep/day. Table 1 shows that OA occurrence, OA diagnosis by a doctor, and current OA treatment status were negatively associated with sleep duration. When adjusting for confounding variables, the significant associations with short sleep duration were maintained in the age-adjusted, sex-adjusted, and fully adjusted analyses for lifetime OA occurrence, diagnosis by a doctor, and current OA occurrence, but not for current OA treatment status (Table 3).

**Table 2:** KL grade and sleep duration, by types of joint.

Factors		Sleep duration						P-value
		Short (≤6 h/day)		Normal (7–8 h/day)		Long (≥9 h/day)		
		N	%	N	%	N	%	
Hip joint KL grade								
	0. Normal	3,362	82.9	3,201	82.18	531	80.2	0.70
	1. Suspected OA	656	16.2	668	17.15	125	18.9	
	2. Mild OA	28	0.7	18	0.46	4	0.6	
	3. Moderate OA	9	0.2	8	0.21	2	0.3	
Knee joint KL grade								
	0. Normal	1,544	38.1	1,681	43.16	214	32.3	<0.0001
	1. Suspected OA	948	23.4	939	24.11	166	25.1	
	2. Mild OA	581	14.3	537	13.79	103	15.6	
	3. Moderate OA	638	15.7	502	12.89	113	17.1	
	4. Severe OA	344	8.5	236	6.06	66	10.0	
Lumbar vertebrae KL grade								
	0. Normal	813	20.1	847	21.75	107	16.2	0.0010
	1. Suspected OA	1,772	43.7	1,816	46.62	301	45.5	
	2. OA	1,470	36.3	1,232	31.63	254	38.4	
OA occurrence								
	No	3,409	84.1	3,462	88.88	539	81.4	<0.0001
	Yes	646	15.9	433	11.12	123	18.6	

Abbreviations: KL grade, Kellgren-Lawrence grade; h, hour; OA, osteoarthritis

**Table 3:** Relationship between sleep duration and OA-related questionnaire items.

Factors			Crude				Adjusted for age and sex				Fully adjusted			
		N (case)	OR	95% CI		P	OR	95% CI		P	OR	95% CI		P
Lifetime OA*														
Sleep	normal	4037 (888)	1.00											
duration†	**short**	4196 (1241)	1.61	1.42	1.81	< .0001	1.30	1.14	1.49	**<0.0001**	**1.28**	**1.12**	**1.47**	**<0.0001**
	long	685 (182)	1.39	1.11	1.74	0.004	1.21	0.95	1.53	0.125	1.18	0.93	1.50	0.168
OA diagnosed by a doctor*														
Sleep	normal	4037 (712)	1.00											
duration†	**short**	4196 (1012)	1.55	1.36	1.77	< .0001	1.21	1.05	1.41	0.009	**1.20**	**1.03**	**1.39**	**0.018**
	long	685 (149)	1.35	1.07	1.70	0.012	1.14	0.89	1.47	0.295	1.11	0.87	1.42	0.406
Current OA*														
Sleep	normal	4037 (599)	1.00											
duration†	**short**	4196 (891)	1.64	1.43	1.89	< .0001	1.29	1.11	1.50	0.001	**1.28**	**1.09**	**1.49**	**0.002**
	long	685 (133)	1.46	1.13	1.87	0.003	1.23	0.94	1.61	0.134	1.18	0.91	1.53	0.213
Currently receiving treatment for OA*														
Sleep	normal	4037 (327)	1.00											
duration†	short	4196 (501)	1.52	1.27	1.82	< .0001	1.15	0.95	1.39	0.164	1.13	0.93	1.36	0.223
	long	685 (84)	1.65	1.19	2.28	0.003	1.32	0.95	1.85	0.103	1.24	0.90	1.72	0.190

Fully adjusted was adjusted for age, sex, household income, education, occupation, marital status, urban-rural gradient, smoking present, body mass index (BMI), and physical activity with a complex sampling design

* Lifetime OA (positive/negative), OA diagnosed by a doctor (yes/no), current OA (positive/negative), currently receiving treatment for OA (yes/no)

†Sleep duration: Normal (7–8 h), Short (≤6 h), Long (≥9 h)

Abbreviations: OA, osteoarthritis; OR, odds ratio; 95% CI, 95% confidence interval.

When we adjusted for confounding variables, there was no significant association between sleep duration and radiological OA diagnosis in the hip joint, knee joint, or the lumbar vertebrae. In the crude analysis, radiological KOA, radiological lumbar OA, and representative OA occurrence showed significant positive associations with short and long sleep duration, but in the age-adjusted, sex-adjusted, and fully adjusted analyses, only representative OA occurrence maintained a significant relationship. Representative OA occurrence, which combines radiological diagnosis and pain, was significantly higher in both the short and long sleep duration groups (Table 4).

**Table 4:** Relationship between sleep duration and radiological OA diagnosis.

Factors			Crude				Adjusted for age and sex				Fully adjusted			
		N (case)	OR	95% CI		P	OR	95% CI		P	OR	95% CI		P
Hip OA diagnosis^a^ (OA_H_scale 0 vs. 2,3)														
Sleep	normal	3289 (29)	1.00				1.00				1.00			
duration†	short	3475 (39)	1.50	0.86	2.62	0.159	1.52	0.84	2.74	0.167	1.48	0.80	2.74	0.209
	long	545 (6)	1.31	0.48	3.55	0.599	1.13	0.41	3.17	0.810	1.31	0.48	3.55	0.599
Knee OA diagnosis^b^ (OA_K_scale 0 vs. 2,3,4)														
Sleep	normal	3028 (1370)	1.00				1.00				1.00			
duration†	short	3182 (1605)	1.40	1.23	1.60	< .0001	1.06	0.91	1.23	0.474	1.07	0.91	1.25	0.426
	long	508 (289)	1.57	1.23	1.99	<0.0001	1.19	0.92	1.55	0.195	1.16	0.89	1.51	0.277
Lumbar OA diagnosis^c^ (OA_L_scale 0 vs. 2)														
Sleep	normal	2085 (1235)	1.00				1.00				1.00			
duration†	short	2288 (1473)	1.31	1.12	1.53	0.001	0.97	0.80	1.16	0.717	0.96	0.80	1.16	0.682
	long	362 (255)	1.47	1.09	1.98	0.012	1.08	0.76	1.52	0.674	0.86	0.60	1.24	0.415
Representative OA occurrence^d^														
Sleep	normal	3988 (441)	1.00				1.00				1.00			
duration†	short	4158 (667)	1.65	1.41	1.92	< .0001	1.23	1.04	1.45	0.015	1.22	1.04	1.44	0.017
	long	676 (126)	1.87	1.43	2.45	< .0001	1.47	1.09	1.98	0.011	1.38	1.03	1.84	0.029

Fully adjusted was adjusted for age, sex, household income, education, occupation, marital status, urban-rural gradient, smoking present, body mass index (BMI), and physical activity with complex sampling design

†Sleep duration: Normal (7–8 h), Short (≤6 h), Long (≥9 h)

^a^Radiological diagnosis of the hip OA: Number of participants with a hip joint Kellgren-Lawrence grade ≥2

^b^Radiological diagnosis of the knee OA: Number of participants with a knee joint Kellgren-Lawrence grade ≥2

^c^Radiological diagnosis of the lumbar vertebral OA: Number of participants with a lumbar vertebrae Kellgren-Lawrence grade of 2 (Lumbar vertebrae Kellgren-Lawrence grade: 0 = normal; 1 = definite osteophyte; 2 = intervertebral disk space narrowing, bone sclerosis, large osteophytes)

^d^Representative OA occurrence: Number of participants with knee joint pain and knee joint Kellgren-Lawrence grade ≥2, or hip joint pain, and hip joint Kellgren-Lawrence grade ≥2

Abbreviations: OA, osteoarthritis; OR, odds ratio; 95% CI, 95% confidence interval.

Table 5 shows the relationship between joint pain and sleep duration for participants with a positive or negative radiological diagnosis for OA of the knee joint. We categorized and compared participants based on their reported pain on the NRS, and analyzed the relationship between pain and sleep duration in more detail. The results are as follows: knee joint pain was significantly associated with sleep duration in participants with a positive radiological diagnosis of KOA only. Participants with knee joint pain with a radiological OA diagnosis and pain (NRS 1–10) had a significant association with **sleep duration** and pain (short sleep duration: OR = 1.32, 95% CI = 1.10–1.58; long sleep duration: OR = 1.41, 95% CI = 1.03–1.95). Participants without a radiological OA diagnosis and pain (NRS 1–10) had no significant association with **sleep duration** and pain. In sub-group analysis according to pain severity, **participants with severe pain (NRS 8–10)** and with/without radiological OA are significantly associated with **short sleep duration** and pain (**OA K scale 0** short sleep duration: OR = 1.82, 95% CI = 1.06–3.11; **OA K scale 2–4** short sleep: OR = 1.34, 95% CI = 1.04–1.73).

**Table 5:** Relationship between sleep time duration and joint pain in adults aged ≥50 years, according to different pain score levels.

NRS score	sleep†		Crude				Adjusted for age and sex				Fully adjusted*			
		N (case)	OR	95% CI		P	OR	95% CI		P	OR	95% CI		P
Pain (1 to 10)	**OA K scale 0**													
	normal	1720 (170)	1.00				1.00				1.00			
	short	1574 (200)	1.42	1.08	1.86	0.012	1.25	0.95	1.64	0.117	1.22	0.92	1.60	0.165
	long	217 (31)	1.56	0.92	2.63	0.100	1.51	0.87	2.63	0.143	1.39	0.80	2.42	0.237
	**OA K scale 2–4**													
	normal	1302 (429)	1.00				1.00				1.00			
	short	1594 (650)	1.45	1.22	1.72	< .0001	1.28	1.07	1.53	0.007	1.32	1.10	1.58	0.003
	long	286 (120)	1.63	1.20	2.22	0.002	1.50	1.10	2.05	0.011	1.41	1.03	1.95	0.034
Mild Pain (1 to 5)	**OA K scale 0**													
	normal	1661 (111)	1.00				1.00				1.00			
	short	1489 (115)	1.23	0.88	1.72	0.236	1.13	0.81	1.58	0.482	1.11	0.79	1.56	0.558
	long	209 (23)	1.88	1.04	3.41	0.036	1.88	1.02	3.46	0.043	1.77	0.97	3.23	0.063
	**OA K scale 2–4**													
	normal	1071 (198)	1.00				1.00				1.00			
	short	1203 (259)	1.34	1.05	1.70	0.017	1.22	0.95	1.56	0.122	1.26	0.98	1.62	0.067
	long	215 (49)	1.64	1.08	2.50	0.022	1.55	1.02	2.37	0.040	1.47	0.96	2.23	0.074
Moderate Pain (6 to 7)	**OA K scale 0**													
	normal	1577 (27)	1.00				1.00				1.00			
	short	1405 (31)	1.33	0.70	2.53	0.388	1.19	0.64	2.20	0.589	1.14	0.62	2.09	0.682
	long	187 (1)	0.33	0.04	2.51	0.282	0.33	0.04	2.51	0.283	0.30	0.04	2.40	0.258
	**OA K scale 2–4 = > NRS4-6**																									
	normal	942 (69)	1.00				1.00				1.00			
	short	1056 (112)	1.40	0.95	2.07	0.094	1.27	0.86	1.88	0.229	1.28	0.86	1.91	0.225
	long	189 (23)	1.40	0.78	2.50	0.256	1.29	0.71	2.35	0.400	1.24	0.68	2.26	0.485
Severe Pain (8 to 10)	**OA K scale 0**													
	normal	1582 (32)	1.00				1.00				1.00			
	short	1428 (54)	2.36	1.40	3.98	0.001	1.86	1.11	3.13	0.019	1.82	1.06	3.11	0.030
	long	193 (7)	1.31	0.50	3.40	0.584	1.05	0.40	2.81	0.917	0.95	0.34	2.67	0.914
	**OA K scale 2–4 = > NRS7-10**													
	normal	1035 (162)	1.00				1.00				1.00			
	short	1223 (279)	1.60	1.24	2.06	**< .0001**	1.35	1.05	1.73	0.021	1.34	1.04	1.73	0.024
	long	214 (48)	1.73	1.17	2.56	0.007	1.48	0.99	2.23	0.059	1.35	0.89	2.05	0.160

*Fully adjusted was adjusted for age, sex, household income, education, occupation, marital status, urban-rural gradient, smoking present, body mass index (BMI), and physical activity with complex sampling design

†Sleep duration: Normal (7–8 hours), Short (≤6 hours), Long (≥9 hours)

Abbreviations: KL grade, Kellgren-Lawrence grade; OR, odds ratio; CI, confidence interval; NRS, Numeric Rating Scale

Table 6 shows the association between sleep duration and pain when adults ≥50 years are classified into three groups: (1) 50–65 years; (2) 65–80; (3) ≥80 years. There was a significant association between short sleep time duration and pain in patients with radiologic OA (K scale 2–4), and among adults aged 50 to 65 years (short sleep: OR = 1.69, 95% CI = 1.18–2.42). Statistically, pain tended to decrease as sleep time duration increased (OR = 0.86, 95% CI = 0.76–0.99).

**Table 6:** Relationship between sleep time duration and joint pain in adults aged ≥50 years, by knee joint KL grade according to age group.

Age group	Sleep	N (case)	Crude				Adjusted for age and sex				Fully adjusted			
			OR	95% CI		P	OR	95% CI		P	OR	95% CI		P
**OA K scale 0**														
Total	normal	1720 (170)	1.00				1.00				1.00			
	short	1574 (200)	1.42	1.08	1.86	0.010	1.25	0.95	1.64	0.120	1.22	0.92	1.60	0.160
	long	217 (31)	1.56	0.92	2.63	0.100	1.51	0.87	2.63	0.140	1.39	0.80	2.42	0.240
50≤ years <65	normal	1318 (121)	1.00				1.00				1.00			
	short	1095 (108)	1.14	0.80	1.63	0.480	1.06	0.74	1.51	0.760	1.05	0.73	1.51	0.810
	long	146 (16)	1.42	0.75	2.68	0.290	1.44	0.75	2.76	0.270	1.36	0.70	2.61	0.360
65≤ years <80	normal	387 (47)	1.00				1.00				1.00			
	short	451 (83)	2.05	1.32	3.19	**< .0001**	1.87	1.19	2.94	0.010	1.94	1.23	3.07	**< .0001**
	long	67 (15)	2.12	0.99	4.54	0.050	2.10	0.90	4.90	0.090	2.20	0.88	5.51	0.090
80≤ *	normal	15 (2)	1.00				1.00				1.00			
	short	28 (9)	3.19	0.41	24.52	0.300	4.29	0.44	42.03	0.200	20.05	0.09	>999.999	0.270
	long	**4 (0)**	<0.001	<0.001	<0.001	< .0001	<0.001	<0.001	<0.001	< .0001	<0.001	<0.001	0.02	**< .0001**
**OA K scale 2–4**														
Total	normal	1302 (429)	1.00				1.00				1.00			
	short	1594 (650)	1.45	1.22	1.72	< .0001	1.28	1.07	1.53	0.010	1.32	1.10	1.58	**< .0001**
	long	286 (120)	1.63	1.20	2.22	**< .0001**	1.50	1.10	2.05	0.010	1.41	1.03	1.95	0.030
50≤ years <65	normal	500 (127)	1.00				1.00				1.00			
	short	502 (154)	1.62	1.15	2.27	0.010	1.51	1.07	2.13	0.020	**1.69**	**1.18**	**2.42**	**< .0001**
	long	73 (26)	1.68	0.90	3.14	0.110	1.83	0.94	3.57	0.080	1.70	0.87	3.35	0.120
65≤ years <80	normal	670 (249)	1.00				1.00				1.00			
	short	955 (428)	1.30	1.02	1.65	0.030	1.19	0.93	1.52	0.170	1.18	0.92	1.50	0.200
	long	174 (75)	1.27	0.88	1.85	0.200	1.30	0.90	1.88	0.170	1.23	0.84	1.81	0.290
80≤	normal	132 (53)	1.00				1.00				1.00			
	short	137 (68)	1.16	0.69	1.96	0.580	1.04	0.60	1.80	0.880	1.09	0.60	1.98	0.770
	long	39 (19)	1.92	0.85	4.32	0.120	1.82	0.81	4.07	0.150	1.97	0.87	4.43	0.100

Fully adjusted was adjusted for age, sex, household income, education, occupation, marital status, urban-rural gradient, smoking present, body mass index (BMI), and physical activity with complex sampling design

†Sleep duration: Normal (7–8 hours), Short (≤6 hours), Long (≥9 hours)

Abbreviations: KL grade, Kellgren-Lawrence grade; OR, odds ratio; CI, confidence interval; NRS, Numeric Rating Scale

## Discussion

This study identified a significant relationship between sleep duration and OA based on the analysis of data from KNHANES-V (2010–2012), a nationwide Korean survey. OA was associated with either short or long sleep duration, rather than normal sleep duration, and the association with short sleep duration was especially strong. However, the association between OA and sleep duration was only observed for participants with a radiological diagnosis of OA and pain; those with pain but no radiological OA diagnosis did not show a significant association between OA and sleep duration. Furthermore, depending on the pain intensity, KOA was associated with short sleep duration, and with short or long sleep duration.

The importance of sleep duration in OA and pain has been emphasized in several previous studies as well as this study. Significant associations have been reported between sleep duration and the prevalence of OA, and over 70% of KOA is accompanied by sleep disorders [32]. Pain provided an explanation of the association between OA and insomnia, and there have been reports that sleep affects pain by various mechanisms [33, 34]. Sanchez et al. found that insufficient sleep quantity may facilitate and/or exacerbate pain through elevations of interleukin (IL)-6 [33]. Quartana et al. announced that sleep disruption is increasingly recognized as a direct contributor to hyperalgesia and impaired endogenous pain modulation [34]. Moreover, in KOA patients, CBT focusing on sleep duration was found to improve pain due to KOA, indicating the importance of sleep in treatment [19]. In people with radiological OA, excessive mechanical load and synovitis are caused during work and activity, which leads to nocturnal knee pain, which reduces sleep quality [35]

Previous studies show that short sleep duration can cause hypertension, glucose intolerance, and cardiovascular disease [9, 11]. Short sleep duration promotes hypertension and cardiovascular disease via increased sympathetic tone and activation of inflammatory pathways [12, 13]. Research has also presented the association between cardiovascular disease and OA prevalence, and that the incidence of hand and knee OA is higher in patients with diabetes than in those without [7, 8]. In summary, shorter sleep duration increases the risk of metabolic disease, which can then increase the incidence of OA as a secondary effect. Consequently, we included behavioral health characteristics that can affect sleep duration or OA (such as chronic disease or mental illness), as potential confounding factors in our study.

Consistent with previous studies, our study revealed an association between OA and short sleep duration [10]. Previous studies reported a high rate of short and long sleep duration among individuals with OA/rheumatism/osteoporosis/arthropathy are all diseases that cause pain; therefore, these diseases can be related to sleep duration, sleep quality, and sleep disorders [36]. This consideration is further supported by reports from individuals with knee or low back pain that is affecting their sleep [17, 37]. Renner et al. [17] found that knee OA was independently associated with increased odds of reporting any sleep problems, such as insomnia (trouble falling asleep, trouble staying asleep, waking early), and insufficient sleep (daytime sleepiness, not enough sleep, not rested). Blake et al. [37] reported adverse sleep effects from chronic lower back pain (LBP), like impaired sleep, sleep disturbances, reduced total sleep time, and an increased number of awakenings and time spent awake after initial sleep onset. Moreover, among knee and LBP patients, higher pain intensity (NRS) affected sleep duration and quality of sleep, demonstrating the importance of pain intensity in sleep disorders [38].

OA can be divided into symptomatic OA (which clinically significant) and radiological OA (which is of diagnostically significant). Studies have shown that clinical pain and radiological evidence of OA are not proportionate in KOA patients. Patients with a high degree of clinical pain but minimal to mild radiographic evidence of joint disease, exhibited greater central sensitization, whereas, patients with low clinical pain with moderate to severe radiographic evidence demonstrated reduced central sensitization [39]. Therefore, we divided our participants into those with and without radiological OA and then performed an analysis by symptomatic OA intensity in each group. Among patients with clinical pain, only a significant relationship with sleep duration in those with radiological OA was observed, and no significant relationship existed with sleep duration in the patients with pain but no radiological OA.

In this study, we demonstrated that sleep duration was related to OA prevalence, and we also found that the relationship between OA and sleep duration only existed for patients with both pain and radiological OA diagnosis. Participants with pain (NRS 1–10) but no radiological OA did not show a significant relationship between OA and sleep duration. However, participants with severe pain (NRS 8–10), short sleep, and pain were associated regardless of the radiological OA (Table 5). However, it is the relationship between pain and sleep duration in patients with OA that explains the structural problems through KL grading and radiological diagnosis. These results show the association between short sleep duration on OA prevalence and the effect of pain on sleep disturbance.

This study utilized large-scale nationwide data from KNHANES to investigate the relationship between sleep duration and musculoskeletal disease. Therefore, the results should have strong explanatory power when applied to the general population; however, there may be limitations regarding generalizability outside the Korean population, and some care is required when interpreting the results. Symptomatic OA, which corresponds to subjective pain and symptoms experienced by the patient, are not always consistent with radiological OA (which refers to abnormalities observed on an X-ray). Consequently, we analyzed both the subjective symptoms and radiological OA prevalence, to elucidate the associations between sleep duration, symptomatic OA (which is clinically important), and radiological OA (which is diagnostically important). In addition, we included numerous health behavioral factors that could affect sleep duration and OA, such as chronic disease or mental illness, as confounding variables. This was done to control the possible associations of chronic disease and mental illness on our results, and accurately investigate the relationship between sleep duration and OA.

As this was a cross-sectional study that utilized data from a national database, we were able to demonstrate a relationship between sleep duration and OA, but could not describe the causal relationship between the two variables. Additionally, we were unable to include information about sleep quality and regularity, which can also indicate sleep disorders; and we only evaluated the presence or absence of pain and its intensity during our pain assessment. The questionnaire did not contain detailed items about the patterns and duration of pain, and though we could account for numerous health-behavioral factors that could affect sleep duration, we could not account for hypnotics or musculoskeletal diseases other than OA, which could also affect sleep duration.

In addition, it was not possible to confirm whether OA diagnosis in the KHANES was a valid statistic that is representative of the Korean population with OA. However, the KHANES is Korea's representative national sample survey, and includes highly reliable data collected by the Korea Centers for Disease Control and Prevention. In particular, the KHANES 5 wave which included radiological examination of OA and KL-grade, a representative diagnostic standard for OA, was used to determine the validity of the diagnosis.

Finally, we have neither dealt with missing data using methods such as multiple imputations (MI) nor adjusted for a more stringent significance threshold, which could only be addressed by a well-designed RCT. However, we believe that our results obtained by excluding the missing data will not differ from the results that we would have obtained if we had processed the missing data using MI. This is because of the nationally representative data used in this study. The stringent significance threshold could be addressed subsequently using well-designed clinical studies.

There have been virtually no studies using large-scale, nationwide data to examine the relationship between sleep duration and OA up until this point. Therefore, this study is of considerable value. Even though several studies have demonstrated the relationship between sleep disorders and joint pain [40, 41], we have added to these findings by investigating how pain intensity and radiological OA affects the relationship between OA and sleep duration. Though we can use our results as a starting point, it will be necessary to perform a prospective study that can elucidate the causal relationship between sleep duration and musculoskeletal disease, and that can include sleep quality. Further research can show the overall pathophysiological relationship between sleep disorders and musculoskeletal diseases, including OA.

## Conclusion

In conclusion, there was a significant association between sleep duration and OA among the general Korean population aged ≥50 years. This study enhanced our understanding of the relationship between sleep duration and OA depending on radiological OA diagnosis, clinical pain (symptomatic OA), and pain intensity. Based on these results, further research will be needed to investigate the pathophysiological relationship between sleep disorders and OA.
